# Botulinum Toxin Injection-Site Selection for a Smooth Shoulder Line: An Anatomical Study

**DOI:** 10.1155/2017/3092720

**Published:** 2017-01-26

**Authors:** Je Hun Lee, Key Youn Lee, Ji Young Kim, Woo Hyeon Son, Ji Heun Jeong, Young Gil Jeong, Seongoh Kwon, Seung Ho Han

**Affiliations:** ^1^Anatomy Laboratory, College of Sports Science, Korea National Sport University, Seoul, Republic of Korea; ^2^College of Veterinary Medicine, Chungnam National University, Daejeon, Republic of Korea; ^3^Department of Anatomy, College of Medicine, Konyang University, Daejeon, Republic of Korea; ^4^Department of Neurosurgery, CHA Gumi Medical Center, CHA University, Pocheon, Republic of Korea; ^5^Department of Anatomy, College of Medicine, Chung-Ang University, Seoul, Republic of Korea

## Abstract

*Introduction.* This study aimed to improve the accuracy of manual needle placement into the trapezius (TM) for smooth shoulder line.* Methods.* For macroscopic study 12 TMs and for microscopic study 4 cadavers were detached and then sampled, 1⁎1 cm at the four points from the origin to insertion site (0% at the most lateral point of external occipital protuberance and 100% at the most lateral point of acromion).* Results.* Most of the nerve endings observed during macroscopic investigations were concentrated in the 60–80% region, and the second most distributed region was the 40–60% region. The microscopic results revealed that the 60–80% region on the reference line had the most dense neuromuscular junction area, while the 40–60% and 80–100% areas were similar in their neuromuscular junction densities.* Discussion.* These anatomical results will be useful in clinical settings especially for cosmetic surgeons.

## 1. Introduction

*Clostridium botulinum* produces an exotoxin that is one of the most poisonous substances in nature. There are several serotypes of* C. botulinum*, A, B, C_1_, C_2_, D, E, F, and G, all of which interfere with neural transmission by blocking the release of acetylcholine at the neuromuscular junction (NMJ) and reduce muscle activity. Among the serotypes, A, B, and F are poisonous to the human nervous system. The US Food and Drug Administration (FDA) approved the use of botulinum toxin-A (BTX), commercially known as Botox, for medical and cosmetic purposes [1–3].

BTX plays a significant role in the management of a wide variety of medical conditions, especially strabismus and focal dystonia, hemifacial spasm, and various spastic movement disorders. Recent research has explored new uses for BTX, including treatment for headaches, hypersalivation, hyperhidrosis, and various chronic conditions that only respond partially to other medical treatments [1, 3]. In two particular studies, injections into the upper trapezius as therapy for myofascial pain syndrome were evaluated [4, 5].

Cosmetic use of BTX increases every year. BTX has mainly been used to correct muscles associated with facial expression on the face. Additionally, Botox injection into the upper side of the trapezius is becoming a common procedure for making a smooth shoulder line. Interest in this procedure is growing but there are few anatomical studies that evaluate innervation effects and optimal needle placement.

The trapezius muscle (TM) is a flat triangular muscle that is one of the major muscles of the back and neck and is responsible for shoulder movement. Angles occur at the shoulder tips, occipital protuberance, and superior nuchal lines, and the inferior angle occurs along the spine at the twelfth thoracic vertebra. The spine portion of the accessory nerve (AN) is innervated and its sensory branches are derived from the ventral rami of C3 and C4. The AN descends from the neck into the deep surface of the TM. The TM helps to elevate, rotate, adduct, and stabilize the scapula and to extend the neck [6].

Studies of TM injections are increasing in number and are yielding important findings [4, 5, 7–9]. For example, one study has shown that BTX injection into the motor end plate region produced significant paralysis and, by moving the injection away from the target region by only 0.5 cm, paralysis decreased by 50%; this indicates that selecting the correct injection point in relation to the most concentrated NMJ site is critical [10–12].

Other studies traced the NMJ on the skeletal muscles of the upper or lower limbs to find the optimal injection point [13–21]. However, no research has evaluated optimal needle placement for injections on the upper border of the TM.

BTX injection can be guided by a variety of techniques, including muscle palpation and location of anatomical landmarks, electrical stimulation, electromyographic guidance (EMG), and ultrasonography. Several studies question whether EMG is really necessary for superficial muscle groups [12, 22, 23], because some patients are unable to cooperate with or tolerate this procedure. In some cases, the operator had to perform the injection without EMG or electric stimulator guidance [12, 22]. In such situations, blind injection was used instead.

Because there have been no studies regarding the location of the optimal NMJ region for injection on the TM, this study aimed to improve the accuracy of manual needle placement into the TM.

## 2. Materials and Methods

### 2.1. Macroscopic Study

Twelve TMs were dissected from 16 human specimens (6 men, 2 women). Any shoulder region showing evidence of prior surgery or injury was excluded. We also excluded any cadavers that showed any evidence of contracture or deformity in the shoulder area.

**Figure 1:**
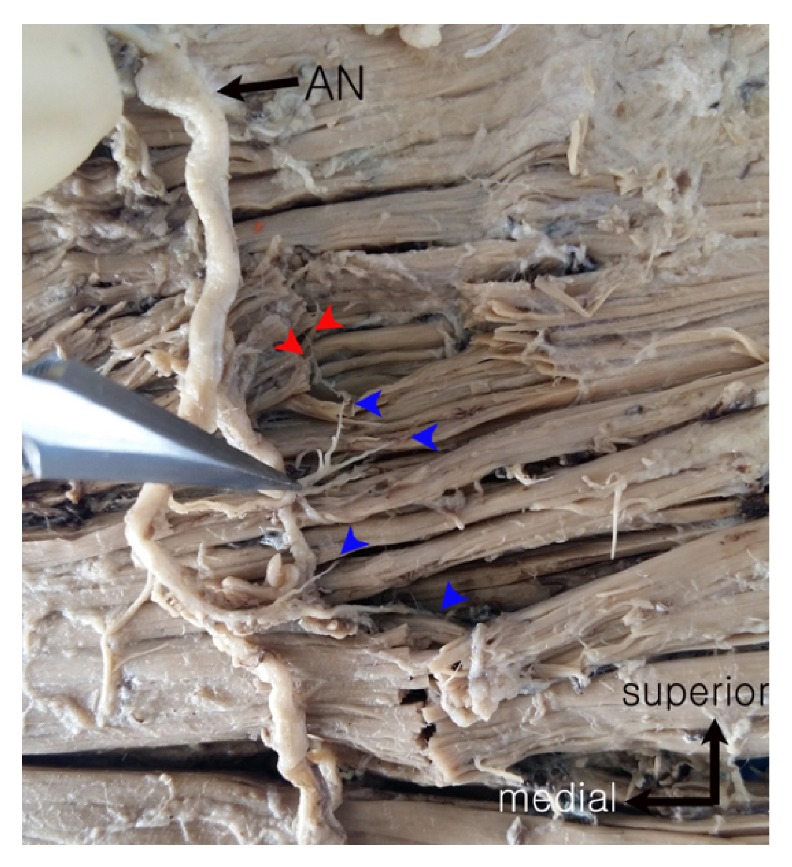
Photographs of the inside of the trapezius muscle where nerve location was traced. AN: accessory nerve; red arrow: intramuscular nerve distribution; blue arrow: nerve entry point into the muscle.

All cadavers were placed in a prone position and facing downward. The superficial fascia and skin were carefully removed and the insertion site was cut. TMs were detached entirely and placed inside out. Since the AN is the main motor nerve to the TM, our dissection focused carefully on tracing the AN until it could be distinguished by the naked eye. We were then able to use an optical surgical microscope (OPMI pico; Carl Zeiss MicroImaging GmbH, Göttingen, Germany) with 3.4–21.3-fold magnification to guide the dissection into the intramuscular regions and terminal ramifications; these anatomical paths were followed as far as they were visible (Figure 1).

**Figure 2:**
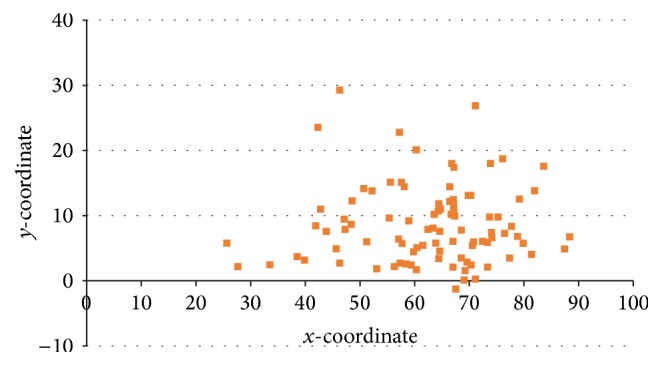
Intramuscular nerve distribution pattern for all specimens based on macroscopic inspection. The *x*-coordinate was the reference line, indicating the most lateral point of external occipital protuberance to the most lateral point of acromion. The *y*-coordinate is perpendicular to the *x*-coordinate according to the measure point (unit: %).

Each motor end plate was marked by pins. We replaced the TM back into the cadaver. The most lateral point of external occipital protuberance became the start point and the most lateral point of the acromion marked the end. As the reference point, we chose the uppermost border of the shoulder. The total length and all end plate points were measured and converted into a percentage; the total length represented 100% of the area (0% at the most lateral point of external occipital protuberance and 100% at the most lateral point of acromion), and each point was converted (point length/total length *∗* 100%) (Figure 2).

### 2.2. Microscopic Study

Four specimens from four cadavers were detached and then sampled, 1*∗*1 cm at the four points from the origin to the insertion site. TMs were divided into four regions: 20–40%, 40–60%, 60–80%, and 80–100% on the reference line. They were carried in 4% PFA, followed by the esterase staining protocol.

For esterase staining, slide samples were placed into a Columbia staining dish. When the staining solution turned gold or red-orange, it was added to the slide sample in the staining dish for 15 minutes at room temperature. After being incubated in the staining solution, slide samples were immediately washed in phosphate buffered saline for several minutes to remove the reaction product. Samples were dehydrated in ascending alcohol solutions in Columbia staining dishes and then cleared with xylene. Slide samples were mounted on a labeled glass slide with mounting solution. Observations were made at five locations on each slide and NMJs were counted using an optical microscope (Leica, magnification 200x).

## 3. Results

**Figure 3 fig3:**
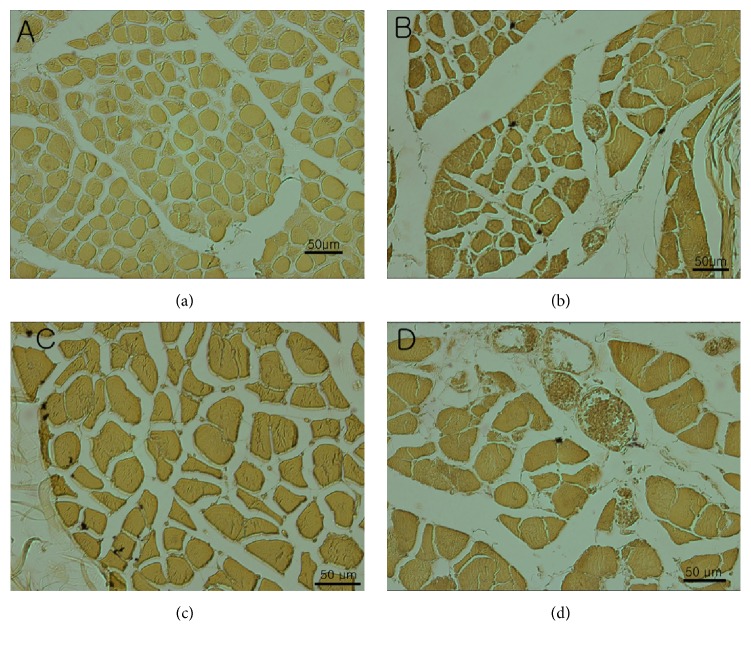
Cryosections from the trapezius muscle of a human specimen. The reference line (100%) indicates the start point, which is the most lateral point of external occipital protuberance and the most lateral point of the acromion. (a) 20–40%; (b) 40–60%; (c) 60–80%; (d) 80–100% of reference line.

A total of 93 points from 16 TMs were evaluated and we divided them into four compartments with 20% intervals on the reference line. Most of the nerve endings observed during macroscopic investigations were concentrated in the 60–80% region (57/93 points, 61.3%), and the second most distributed region was the 40–60% region (27/93 points, 29.0%). All other regions were occupied at very low percentages (Table 1 and Figure 3).

**Figure 4:**
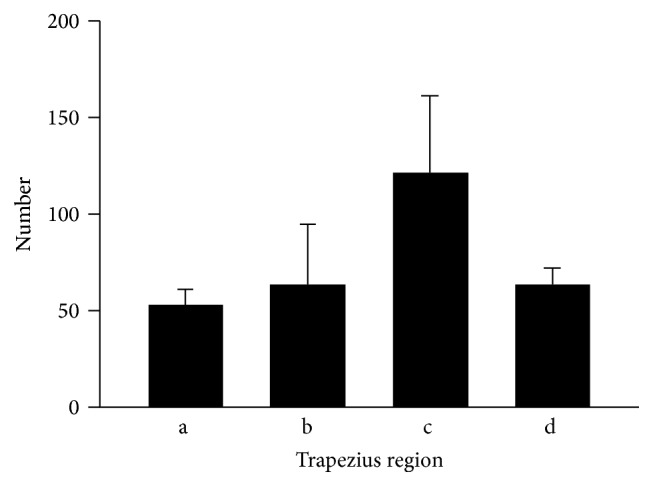
The quantity of neuromuscular junctions based on esterase staining. The *x*-coordinate is the reference line and indicates the most lateral point of external occipital protuberance to the most lateral point of acromion. (a) 20–40%; (b) 40–60%; (c) 60–80%; (d) 80–100% of reference line.

The esterase staining results revealed that the 60–80% region on the reference line had the most dense NMJ area, while the (b) (40–60%) and (d) (80–100%) areas were similar in their NMJ densities. The (a) area had the smallest density of all the regions (Figures 3 and 4).

## 4. Discussion

The role of BTX in various facial treatments is well known. For medical purposes, it has become an important treatment for facial disorders, including focal dystonia, hemifacial spasm, and various spastic movement disorders [1]. However, its use is not limited to facial treatments. It is also used for motor or sensory disorders related to abnormal muscle activity on the upper or lower limbs. Poststroke spasticity [24] or hemiplegic cerebral palsy in children [25] involves problems with the upper flexor muscles. Supraspinal spasticity can present as disorders of the flexor pattern of the hips and knees [26]. Meanwhile, other clinical research has identified value of BTX for treating headaches, hypersalivation, hyperhidrosis, and other chronic conditions that respond only partially to medical treatment [1]. These reports are encouraging for further research into the use of BTX on the TM. Many new uses of Botox involve the upper shoulder border for controlling myofascial pain syndrome and other related pains, such as chronic neck or back pain, cervicogenic headache, and focal dystonia of the shoulder [4, 5, 7–9]. Even though reports of successful treatments are increasing in frequency, we still do not have a clear understanding of the underlying mechanism. Our study moves this research a step forward in terms of understanding pain control through BTX injections in the TM.

Additionally, for aesthetic purposes, BTX has been used to improve wrinkles and correct the muscles involved in facial expression [1]. Along with facial procedures, in Korea, brides are interested in receiving BTX on their TM, at the upper border of the trapezius, to create a smooth shoulder line for their wedding dress. Since this cosmetic procedure requires delicacy and control, guidelines for needle placement are important for successful outcomes.

Because BTX can be used for managing various spasticities, many studies have explored the safety and effectiveness of this treatment. Since BTX is one of the most poisonous substances, toxin dosage and its dilution ratio have been actively studied. Shaari and Sanders showed the importance of injecting BTX directly into the NMJ region of a muscle, since injections placed only 5 mm away from the NMJ resulted in a 50% decrease in paralysis [27]. Mayer et al. observed that injecting a single motor point and multisite distributed injections had similar effects [28]. Increases in dosage increased paralysis strength; however, delivering toxins in small volumes directly into the NMJ region produced the most effective paralysis [27]. Since there has been no previous research on TM injections, blind injections, which are inexact, have been performed. Even though EMG can improve accuracy, some patients dislike it and it is often not available [12, 22, 23]. Additionally, some studies suggest that if the target muscle is part of a superficial muscle group, injection with palpation is recommended [12, 22]. Since TM fully qualifies for blind injection, a concentrated NMJ region should be a valuable guide.

Recently, some studies [29, 30] described the use of an esterase stain on an animal sample. This is the best microscopic anatomical method to investigate the NMJ distribution on a muscle. We experienced some trial and error because of cadaver conditions and with histologic processing. However, we used two research methods (macro and micro) and found common results showing that the area from 60 to 80% on the reference line had the highest density of NMJs (Table 1, Figures 2 [Fig fig3]–4). These anatomical results will be useful in clinical settings.

## 5. Conclusion

Based on the results from this study, we recommend that for the TM botulinum injections be placed between 60.0% and 80.0% distance of the reference line from the most lateral point of external occipital protuberance to the most lateral point of acromion. The results of this study may aid in the more accurate localization of the ideal injection sites for smooth shoulder line and better guarantee maximal botulinum efficacy.

## Figures and Tables

**Table 1:** The distribution pattern of neuromuscular junctions according to macroscopic investigation.

Region (%)	Points	Percentage (%)
20–40	4	4.3%
40–60	27	29.0%
60–80	57	61.3%
80–100	5	5.4%
Total	93	100%
